# Retraction: 5-aminosalicylic acid improves lipid profile in mice fed a high-fat cholesterol diet through its dual effects on intestinal PPARγ and PPARα

**DOI:** 10.1371/journal.pone.0200947

**Published:** 2018-07-12

**Authors:** Zheng Wang, Debby Koonen, Marten Hofker, Zhijun Bao, the PLOS ONE Editors

After publication of this work [1], *PLOS ONE* was notified of several concerns:

- The second and third authors (DK, MH) were not aware of and did not approve this submission to *PLOS ONE*; MH passed away in 2016, before this manuscript was submitted to *PLOS ONE*. Incorrect email addresses for these authors were provided to the journal when the manuscript was submitted.
- Questions were raised as to whether the corresponding author had appropriate permissions to publish the data, and whether author contributions had been accurately reported.
- The research reported in Figs 1–3 and the qPCR data for PPARα and PPARγ were reported in a previously published article [2] that was not cited or discussed in [1].

In light of these concerns, the authors and *PLOS ONE* Editors retract this article [1].

ZW, DK, and ZB agreed with the retraction.
